# *GAD2* Alternative Transcripts in the Human Prefrontal Cortex, and in Schizophrenia and Affective Disorders

**DOI:** 10.1371/journal.pone.0148558

**Published:** 2016-02-05

**Authors:** Kasey N. Davis, Ran Tao, Chao Li, Yuan Gao, Marjorie C. Gondré-Lewis, Barbara K. Lipska, Joo Heon Shin, Bin Xie, Tianzhang Ye, Daniel R. Weinberger, Joel E. Kleinman, Thomas M. Hyde

**Affiliations:** 1 Clinical Brain Disorders Branch, Genes, Cognition and Psychosis Program, Intramural Research Program, National Institute of Mental Health, National Institutes of Health, Bethesda, Maryland, 20892–1385, United States of America; 2 The Lieber Institute for Brain Development, Johns Hopkins University School of Medicine, Baltimore, Maryland, 21205, United States of America; 3 Department of Psychiatry and Behavior Sciences, and Neurology, Johns Hopkins University School of Medicine, Johns Hopkins University Medical Campus, Baltimore, Maryland, 21205, United States of America; 4 Departments of Neuroscience and the Institute of Genetic Medicine, Johns Hopkins University School of Medicine, Baltimore, Maryland, 21205, United States of America; 5 Laboratory for Neurodevelopment, Department of Anatomy, Howard University College of Medicine, Washington D.C., 20059, United States of America; UTHSCSH, UNITED STATES

## Abstract

Genetic variation and early adverse environmental events work together to increase risk for schizophrenia. γ-aminobutyric acid (GABA), the major inhibitory neurotransmitter in adult mammalian brain, plays a major role in normal brain development, and has been strongly implicated in the pathobiology of schizophrenia. GABA synthesis is controlled by two glutamic acid decarboxylase (GAD) genes, *GAD1* and *GAD2*, both of which produce a number of alternative transcripts. Genetic variants in the *GAD1* gene are associated with increased risk for schizophrenia, and reduced expression of its major transcript in the human dorsolateral prefrontal cortex (DLPFC). No consistent changes in *GAD2* expression have been found in brains from patients with schizophrenia. In this work, with the use of RNA sequencing and PCR technologies, we confirmed and tracked the expression of an alternative truncated transcript of *GAD2* (ENST00000428517) in human control DLPFC homogenates across lifespan besides the well-known full length transcript of *GAD2*. In addition, using quantitative RT-PCR, expression of *GAD2* full length and truncated transcripts were measured in the DLPFC of patients with schizophrenia, bipolar disorder and major depression. The expression of *GAD2* full length transcript is decreased in the DLPFC of schizophrenia and bipolar disorder patients, while *GAD2* truncated transcript is increased in bipolar disorder patients but decreased in schizophrenia patients. Moreover, the patients with schizophrenia with completed suicide or positive nicotine exposure showed significantly higher expression of *GAD2* full length transcript. Alternative transcripts of *GAD2* may be important in the growth and development of GABA-synthesizing neurons as well as abnormal GABA signaling in the DLPFC of patients with schizophrenia and affective disorders.

## Introduction

GABA, the major inhibitory neurotransmitter in mammalian brain, has been implicated in both brain development and schizophrenia [1–3]. Two genes, *GAD1* and *GAD2*, control GABA synthesis, but only the former has been clearly implicated in schizophrenia [4,5]. *GAD1* and *GAD2* are located on different chromosomes in mammals and encode two major isoforms of the enzyme glutamate decarboxylase (GAD), GAD67 and GAD65 respectively [6]. Within cells, GAD2 full length protein (65kDa) is maintained in a largely inactive form, apoGAD, (approximately 93%), which is converted to an enzymatically active form through the binding of pyridoxal 5’-phosphate. In contrast, GAD1 full length protein (67kDa) is found mainly as holoGAD (approximately 72%), which is enzymatically active and continually produces GABA [7]. Studies of *GAD2* in postmortem brains of patients with schizophrenia have been inconsistent and mostly negative. In prefrontal cortex (PFC), *GAD2* expression has been reported as decreased [8], increased [9] and normal [10,11] in patients with schizophrenia.

The GAD2 and GAD1 full length proteins have quite different distributions and functions. Located predominantly in synapses, GAD2 full length protein is associated with synaptic vesicles and produces synaptic GABA during intense neuronal activity [12]. GAD1 full length protein, on the other hand, can be found in somatic-dendritic regions as well as the synaptic terminal [13]. Studies in transgenic mice suggest additional distinct roles of these two GAD isoforms. Homozygous *GAD1* knockout mice die at birth and have 10% of the normal GABA content, whereas *GAD2* knockout mice are viable and have normal GABA content at birth [14,15]. Although viable, *GAD2* deficient mice are susceptible to seizures, show a reduction in GABA release during prolonged activation of inhibitory neurons, and decreased GABA release in the visual cortex with potassium stimulation [14,16,17]. Interestingly, *GAD2* knockout mice exhibit deficits in prepulse inhibition, an abnormality involving defective modulation of the startle reflex, also associated with schizophrenia [18]. These findings support the notion that *GAD2* plays a role in the synthesis of GABA for synaptic release.

Previous work from our group has demonstrated the importance of alternate transcripts from *GAD1* in both early brain development and schizophrenia [19]. The characterization of *GAD2* alternative transcripts has not been completed. In order to study *GAD2* thoroughly in brains of patients with schizophrenia we have done the following experiments: 1. Identified alternate transcripts in postmortem human brain; 2. Measured the expression of *GAD2* transcripts from prenatal week 14 through 80 years of age in human prefrontal cortex (PFC); and 3. Compared the expression of *GAD2* transcripts in dorsolateral prefrontal cortex (DLPFC) of patients with schizophrenia, affective disorders and normal controls.

## Materials and Methods

### Human postmortem brain tissue collection, tissue retrieval and RNA extraction

Postmortem neonate, infant, child, adolescent and adult brains were collected at the Clinical Brain Disorders Branch (CBDB), National Institute of Mental Health (NIMH) through the Northern Virginia and District of Columbia Medical Examiners’ Office. Informed consent to study brain tissue was obtained from the legal next of kin for all cases, according to NIMH Protocol 90-M-0142 and processed approved by the NIMH/National Institutes of Health Institutional Review Board [20]. Additional fetal, child and adolescent brain tissue samples were provided by the National Institute of Child Health and Human Development Brain and Tissue Bank for Developmental Disorders under contacts N01-HD-4-3368 and NO1-HD-4_3383, approved by institutional review board of the University of Maryland at Baltimore and the State of Maryland, and the tissue was donated to the NIMH under the terms of a material transfer agreement. All samples were obtained with audio-taped informed consent from the legal next of kin to study brain tissue, as approved by the Institutional Review Board of the National Institutes of Health and University of Maryland, in lieu of a written consent form. Brains were hemisected, and cut into 1.0–1.5-cm-thick coronal slabs, flash frozen, and stored at -80°C. DLPFC gray matter was dissected using a dental drill. For CBDB cases, the DLPFC (Brodmann’s areas 9 and 46) were dissected from middle frontal gyrus from coronal slab immediately anterior to the genu of the corpus callosum. For fetal cases, the PFC was obtained from the frontal cortex dissected at the dorsal convexity, midway between the frontal pole and anterior temporal pole. To measure the pH, pulverized cerebellum was used from each sample. To assess the expression of *GAD2* alternative transcripts in the DLPFC, 176 schizophrenia patients, 61 bipolar disorder patients, 138 major depressive disorder (MDD), and 364 non-neurological and non-psychiatric controls were studied. A subset of non-psychiatric non-neurological controls, spanning from gestational weeks 14 to 20 and from birth up to 85 years of age, were used to measure expression of selected *GAD2* transcripts in the prefrontal cortex (Table 1). Toxicological analysis was performed on all samples. Positive toxicology was exclusionary for control subjects but not for patients with psychiatric disorders. The majority of positive toxicology reports in psychiatric cases were due to the presence of psychotropic medications. In a subset of psychiatric cases, toxicology studies also revealed the presence of a variety of illicit substances. Nonpsychiatric controls were excluded if toxicology was positive for psychotropic medications or illicit substances. 43.6% of psychiatric patients (63.8% in MDD, 65.6% in bipolar disorder, and 20% in schizophrenia) are suicides. Nicotine exposure was positive in 41.8% patients with psychiatric disorders (43.4% in schizophrenia, 43.7% in bipolar disorder and 35.1% in MDD) and 24.6% controls.

**Table 1:** Demographic information of our human postmortem samples.

Cohort	Number	Race	Sex	Age	PMI(h)	pH	RIN
Controls	326	117AA/107CAUC/6HISP/6AS	167M/69F	41±17.1	29.3±14.5	6.55±0.28	8.30±0.69
SZ patients	176	73AA/96CAUC/4HISP/3AS	111M/65F	50±15	38.6±24.1	6.40±0.25	7.84±0.96
BP patients	61	6AA/51CAUC/1HISP/3AS	36M/25F	45±14	32.9±18.4	6.36±0.28	7.99±0.86
MDD patients	138	14AA/119CAUC/3HISP/2AS	79M/59F	45±14	37.8±28.3	6.35±0.28	8.03±0.88
**Lifespan**							
Fetal Controls	43	37AA/5CAUC/1HISP	22M/21F	-0.42±0.07^*a*^	2.58±2.14	N/A	8.81±1.29
Postnatal Controls	283	139AA/132CAUC/6HISP/6AS	198M/85F	34.99±20.84	28.9±14.4	6.52±0.30	8.22±0.79

AA, African American; CAUC, Caucasian; AS, Asian; HISP, Hispanic; F, Female; M, Male; Sz, Schizophrenia; BP, Bipolar Disorder; MDD, Major Depressive Disorder; PMI, Postmortem Interval; RIN, RNA Integrity Number.

^a^Fetal cohort in gestational age in weeks.

Dissected tissue was pulverized and stored at -80°C; RNA extractions and reverse transcriptase reactions were performed as described previously [20]. Briefly, total RNA was extracted from 300mg of tissue with TRIZOL Reagent (Life Technologies, Grand Island, New York). The yield of total RNA was determined by measuring the absorbance at 260 nm. To generate a RNA Integrity Number (RIN), RNA quality was assessed by high-resolution capillary electrophoreses (Agilent Technologies, Palo Alto, California). Using SuperScript First-Strand Synthesis System for RT-PCR (Invitrogen, Carlsbad, CA, USA) to synthesize cDNA, 4μg of total RNA was reverse transcribed in a 50μl reaction system.

### RNA Sequencing

To identify the library of transcripts derived from the *GAD2* gene, RNA sequencing was performed on pooled commercial fetal and adult whole human brain poly A^+^ RNAs (Clontech, Lot#7110099, Lot#110415). mRNA molecules were fragmented into small pieces using divalent cations under elevated temperatures. Reverse transcriptase and random hexamers were used to convert the cleaved RNA fragments into first strand cDNA. Second strand cDNA was synthesized using DNA Polymerase I and RNaseH. These cDNA fragments were then subjected to an end repair process using T4 DNA polymerase, T4 polynucleotide kinase (PNK) and Klenow DNA polymerase, the addition of a single ‘A’ base using Klenow exo (3’ to 5’ exo minus), and then ligation of the Illumina P adapters using T4 DNA Ligase. An index (up to 12) was inserted into Illumina adapters so that multiple samples could be sequenced in one lane of an 8-lane flow cell if necessary. These products were then purified and enriched by PCR to create the final cDNA library for high throughput DNA sequencing using HiSeq 2000 (Illumina, Inc.). To analyze the RNASeq data, sequencing reads were mapped against the whole reference genome by using TopHat software. The Cufflinks software was used to assemble transcripts and estimate their abundance.

### Rapid amplification of cDNA (RACE) ends and end-to-end PCR

To identify the 3’ ends of *GAD2* transcript in human brain, we performed 3’ RACE, using fetal and adult brain poly A+ RNA with *GAD2* gene specific sense primers binding at exon 1. We used the SMART RACE cDNA Amplification Kit (Clontech) and Advantage 2 PCR Kit (Clonetech) for these assays. Commercial human fetal and adult brain poly A+ RNAs (Clontech) were reverse-transcribed to cDNA by MMLV reverse transcriptase (Clontech) according to the manufacturer’s protocol. The PCR amplification profile was 94°C for 2 min, 45 cycles of 94°C for 30 s, 68–72°C for 30s, 68°C for 2–6 min, and 68°C for 10min after the last cycle. Based on known *GAD2* gene exons (NM_001134366.1) and 3r RACE results, we designed primer pairs to amplify full length and portion of *GAD2* transcripts using Platinum TaqDNA polymerase (Invitrogen). The PCR conditions were 94°C for 3 minutes, 30 cycles of 94°C for 30 sec, 56°C for 30 sec, 72°C for 1–5 minute, and 72°C for 10 minutes after the last cycle. RACE and end-to-end PCR products were cloned into E. Coli by PCR-TOPO 4.0 vectors (Invitrogen^TM^) and sequenced. All PCR results were confirmed in separate PCR assays and Sanger sequencing.

### DNA extraction and sequencing

For extraction of DNA from E. coli, the Qiagen QlAprep spin miniprep kit (QIAGEN Science, Maryland, USA) was used. DNA was sent for sequencing at the National Institute of Neurological Disorders and Strokes (NINDS), using Big Dye Terminators on an ABI Perkin Elmer 9700 Thermal Cycler according to the manufacturer’s protocol. DNA was then sequenced using an Applied Biosystem 3100 Genetic Analyzer. The final data was analyzed using the Applied Biosystems Sequence Scanner V.1.0.

### Quantitative real-time PCR

mRNA expression levels of alternative *GAD2* transcripts were measured in postmortem DLPFC samples, including 417 non-psychiatric non-neurological control subjects, 176 schizophrenia patients, 61 bipolar disorder patients, and 138 major depression patients by real-time quantitative (RT-PCR), using ABI Prism 7900 sequence detection system with 384-well format. TaqMan Gene Expression Assays (Applied Biosystems, Foster City, CA, USA) were used; for human *GAD2* (spanning exons 15–16, Hs00609534_m1); for human *GAD2* truncated transcript (spanning exons 4 a-intron4; Table 2). mRNA expression levels of two *GAD2* alternative transcripts (*GAD2* full length transcript and truncated transcript) were normalized to the geometric means of three constitutively-expressed genes: β-actin (ACTB), β2-microglobulin (B2M) and β-glucuronidase (GUSB). The geometric means of the three housekeeping genes did not show diagnostic group difference. Genotypes were generated as described previously by Straub et al [21].

**Table 2:** QPCR Primers and Probes.

Transcript	Primers and Probes
ENST00000428517	Forward: AATTGGGAATTGGCAGACCA
	Reverse: ATTTTCGATGAGACAATACCTGTTT
	Probe: TTGATGCATTGCCAAACA

### Statistical analysis

Statistical analyses were performed using R package (Version3.2.2). The expression data was log2 transformed to make the data more symmetrical for ANCOVA/linear modeling. Levene's test showed that the assumption of homogeneity of variance was met. The lifespan curve was generated using LOESS fit (local polynomial regression fitting) by using an R package with default parameters. ANCOVA/linear model was used for all comparisons. Multiple regression analyses were carried out to evaluate the contributions of age, pH, postmortem interval (PMI), race, sex and RNA integrity number (RIN), ethanol exposure and nicotine exposure on mRNA expression. Multiple regression analyses were also performed to assess the effect of completed suicide, psychotropic medications (toxicology screen results about antidepressants, antipsychotics, anticonvulsants, benzodiazepines, and opiates), lifetime neuroleptic exposure, average daily neuroleptic dose, final neuroleptic dose, and illicit substances on mRNA expression in the patients with psychiatric disorders. Estimated of lifetime neuroleptic exposure, average daily dose and final neuroleptic dose were all converted to chlorpromazine equivalents for statistical comparisons. Comparisons between diagnostic groups were made by ANCOVA for mRNA expression with diagnosis as an independent variable. Covariates were chosen for each ANCOVA from multiple regression analyses. Comparisons between patients and controls following overall ANCOVA were conducted by post-hoc Tukey HSD tests. Comparisons within the different diagnostic groups for completed suicide, nicotine exposure, ethanol exposure and psychotropic medications were conducted by ANCOVA, followed by Bonferroni correction.

## Results

### Alternative transcripts of *GAD2* in human brain

**Fig 1:**
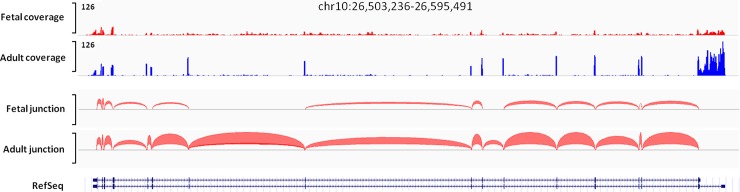
Mapped Reads and Junction Plots from RNA sequencing of *GAD2*. Using pooled whole brain, known and putative novel exons for *GAD2* in both adult and fetal human brain were identified using RNA-Seq. The y-axis is the coverage information, while the x-axis shows the position of the reads. The red arc-shapes between exons represented the splicing junctions. The thickness of the red arc-shapes represented the abundance of the splicing events. We observed the continuously splicing events across all the known junction of the *GAD2* gene in adult brain but not in fetal brain. These results suggested there isn’t a novel splicing junction for *GAD2* in the human DLPFC, and potential truncated transcripts composed by several 5’ exons.

An analysis of pooled RNA from normal fetal or adult brains (acquired commercially) by RNA sequencing failed to identify any novel splicing junctions of *GAD2* in the human DLPFC, but confirmed the production of a previously identified truncated transcript (ENST00000428517) composed of several 5’ exons of *GAD2* (Fig 1).

**Fig 2:**
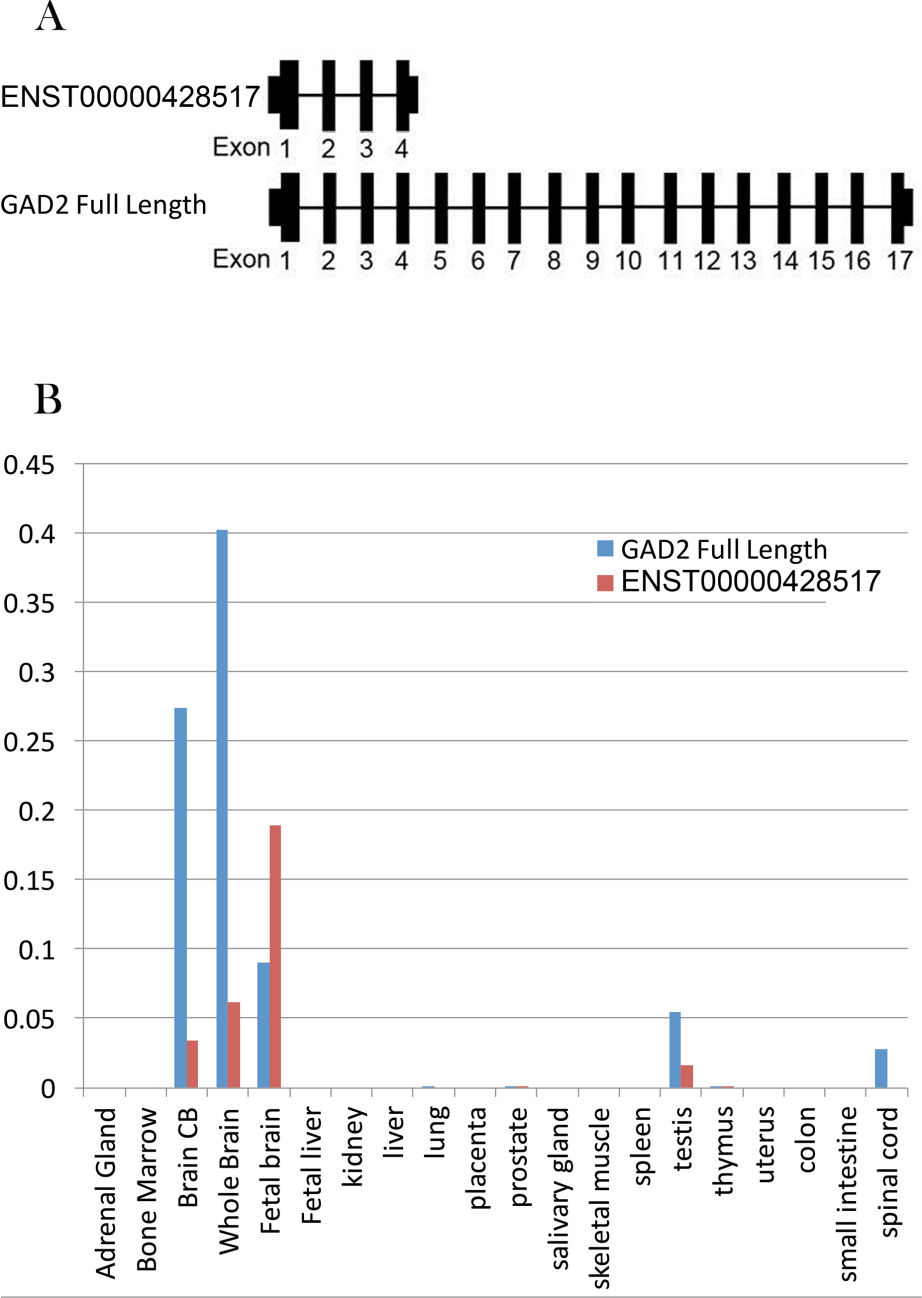
Alternative transcript map for *GAD2*. (A) Alternative transcripts of *GAD2* in human Brain. Full length *GAD2* transcript (NM_001134366.1, encoding the 65kDa GAD protein) and a truncated transcript (ENST00000428517) were identified. Key: Black rectangles, known exons. (B) The expression of *GAD2* transcripts in 20 human tissues. *GAD2* were mainly expressed in human brain. These two transcripts had opposite developmental expression changes in human brain. All the expression levels were normalized by ACTB, B2M and GUSB.

To validate the findings from the RNA-Seq data, we carried out 3’RACE and observed an alternative termination point with a poly A+ tail in intron 3. By end-to end PCR, we confirmed the well-known full length transcript encoding for the 65kDa GAD protein and the previously identified truncated transcript, ENST00000428517, consisting of the first four exons of *GAD2* (Fig 2A). RNA sequencing data suggested that the expression of the *GAD2* truncated transcript is about 78% of the expression of the full length *GAD2* in the fetal brain, about 12% in the childhood (0~10 years old) and 4% after 10 years old [22]. The expression levels of the full length and truncated *GAD2* transcripts in 20 pooled human tissues (acquired commercially from Clontech, Human Total RNA Master Panel II, Lot#1403130A) showed that both *GAD2* transcripts are primarily expressed in brain (Fig 2B). The truncated transcript shares the same exon1 and translational start site with the full length transcript, but lacks an in-frame stop codon.

### Lifespan expression pattern of *GAD2* alternative transcripts

**Fig 3:**
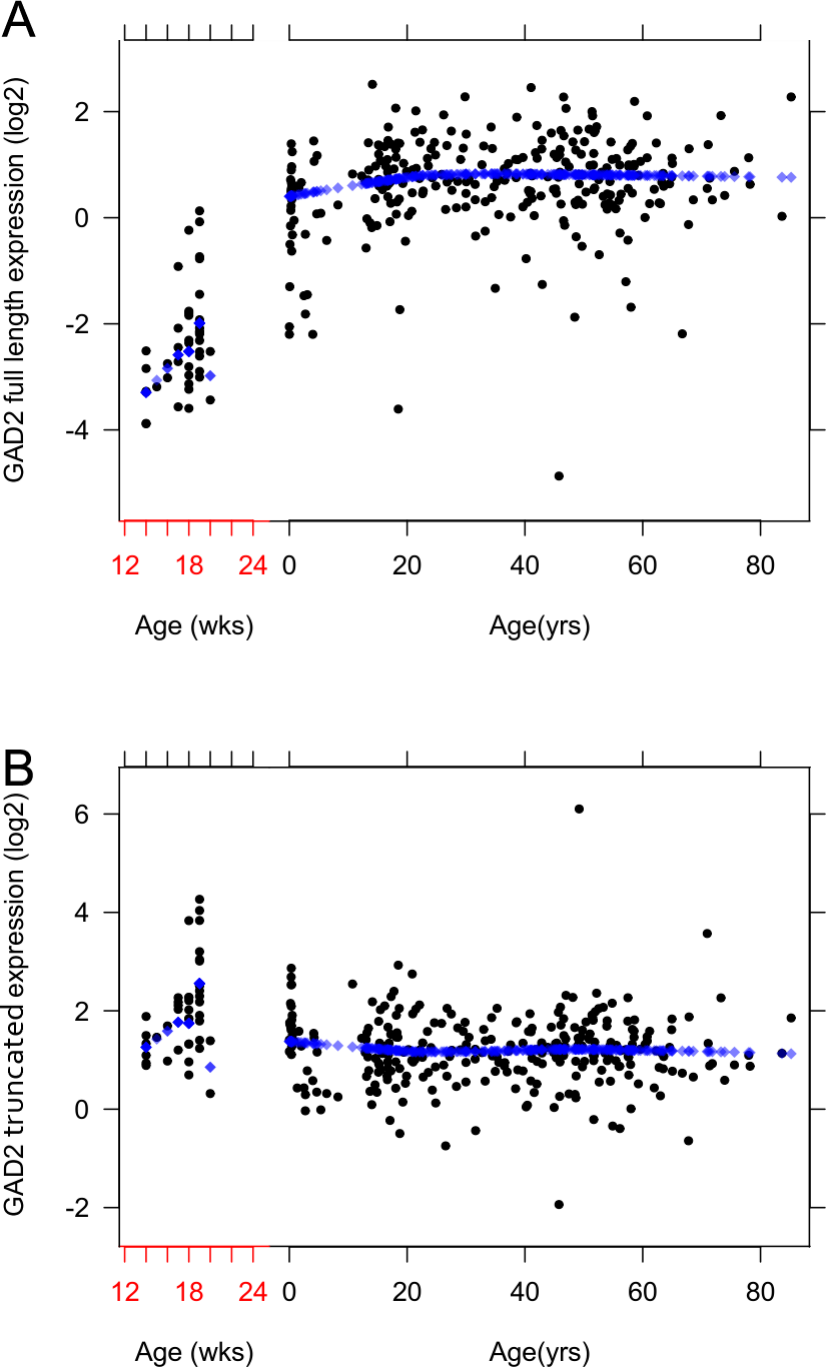
Lifespan expression of the *GAD2* alternative transcripts. Each figure shows the expression patterns of the selected *GAD2* alternative transcripts across the lifespan in the human DLPFC: (A) The expression of *GAD2* full length across the lifespan in the human DLPFC. (B) The expression of ENST00000428517 across the lifespan in the human DLPFC. The expression of full length *GAD2* transcript increases from 14 weeks of gestational age until the first decade of life and levels off throughout the rest of the lifespan. ENST00000428517 expression in the DLPFC is highest in the fetus and then declines gradually. The x-axis shows age: Before birth gestational age is in weeks (wks) and after birth in years (yrs). On the y-axis, the expressions levels were Log2 normalized. Each dot represents an individual subject.

In a cohort of 326 non-neurologic non-psychiatric control subjects ranging from the second trimester in the fetus through 85 years of age, prefrontal cortical samples were studied to determine the expression pattern of the two transcripts across the human lifespan. Unlike the lifespan pattern of the *GAD2* full length transcript (Fig 3A), whose expression was low in the fetus and then gradually rose to its adult peak at about 20 years of age, the *GAD2* truncated transcript, ENST00000428517, showed a mildly inverted expression pattern. ENST00000428517 expression peaked during fetal period, and then gradually decreased after birth (Fig 3B).

### Expression of *GAD2* splice variants RNA in schizophrenia, bipolar disorder and major depression

**Fig 4:**
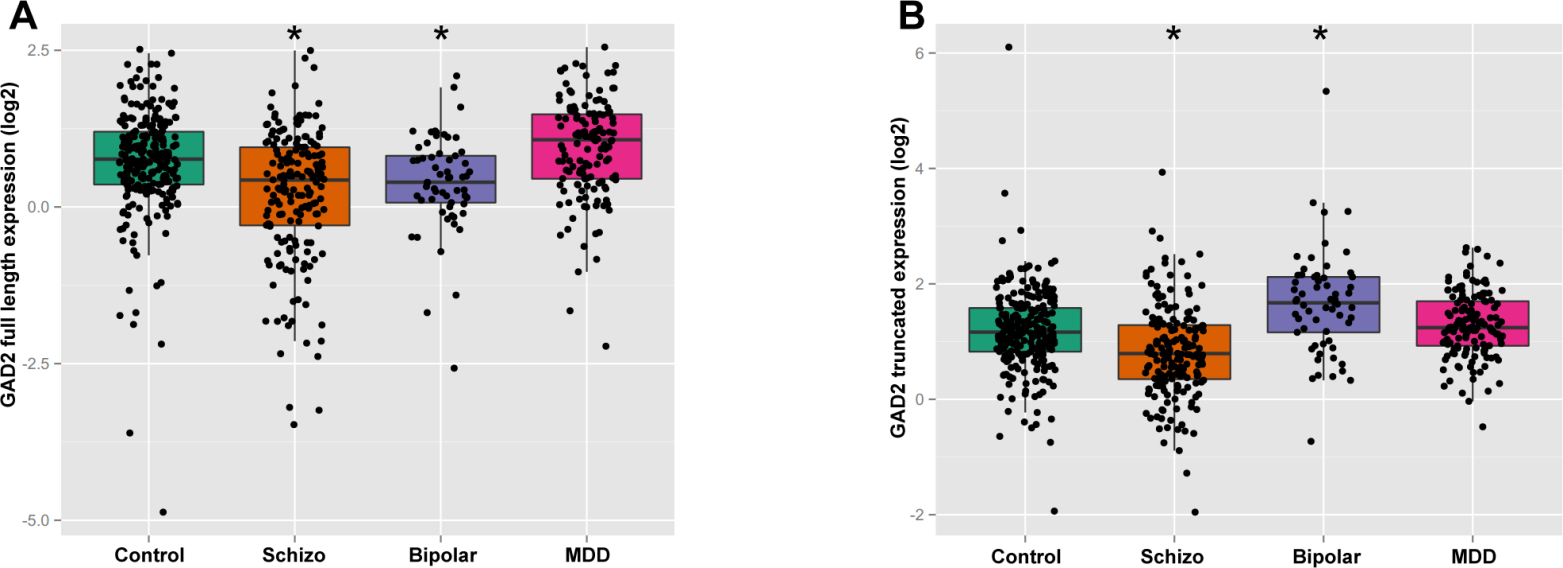
Alternative *GAD2* transcript expression in four diagnostic groups. The expression of each alternative transcript was studied across four cohorts of subjects (non-psychiatric controls, schizophrenia, bipolar disorder, and major depressive disorder). Each panel shows the comparison of each transcript across the groups: (A) *GAD2* full length in DLPFC; (B) ENST00000428517 in DLPFC. The x-axis shows the different diagnostic groups: Control, nonpsychiatric control group; Schizo, patients with schizophrenia; MDD, patients with major depressive disorder. The y-axis represents relative expression in the DLPFC. The y-axis is the least-squares means of expression with log2 normalization, computed for covariates at their means. Asterisks mark symbolizes statistically significance between psychiatric group and control group. Each dot represents an individual subject.

Multiple regression analyses revealed that the mRNA expression level of *GAD2* full length transcript positively correlated with age (p = 2.71E-05), sex (p = 1.96E-08), RIN (p<2E-16), pH (p = 1.19E-10) and PMI (P = 9.2E-03) in the DLPFC. The mRNA expression of ENST00000428517 was positively correlated with PMI (p = 1.60E-02) and sex (p = 1.94E-03) in the DLPFC. Each positive variable was included as covariates in ANCOVA. An ANCOVA revealed that in the DLPFC, the expression of *GAD2* full length showed an overall effect of diagnosis (F_(3,610) =_ 26.67,p = 3.22E-16; Fig 4A). Post-hoc analyses revealed that the expression of *GAD2* full length was significantly decreased in schizophrenia patients (24% decrease, p = 7.05E-10) and bipolar disorder patients (22% decrease, p = 1.28E-02), with no changes in MDD patients (p = 5.92E-02) compared with controls. Regarding the truncated transcript ENST00000428517, there was also an overall effect of diagnosis (F_(3,613) =_ 23.17,p = 3.21E-14; Fig 4B). Patients with bipolar disorder exhibited a 48% increase in ENST00000428517 mRNA expression (p = 5.76E-06), patients with schizophrenia (p = 5.76E-06) showed a 24% decrease, while MDD (p = 0.55) patients showed no difference.

### Expression of *GAD2* full length transcript with nicotine exposure and suicide

**Fig 5:**
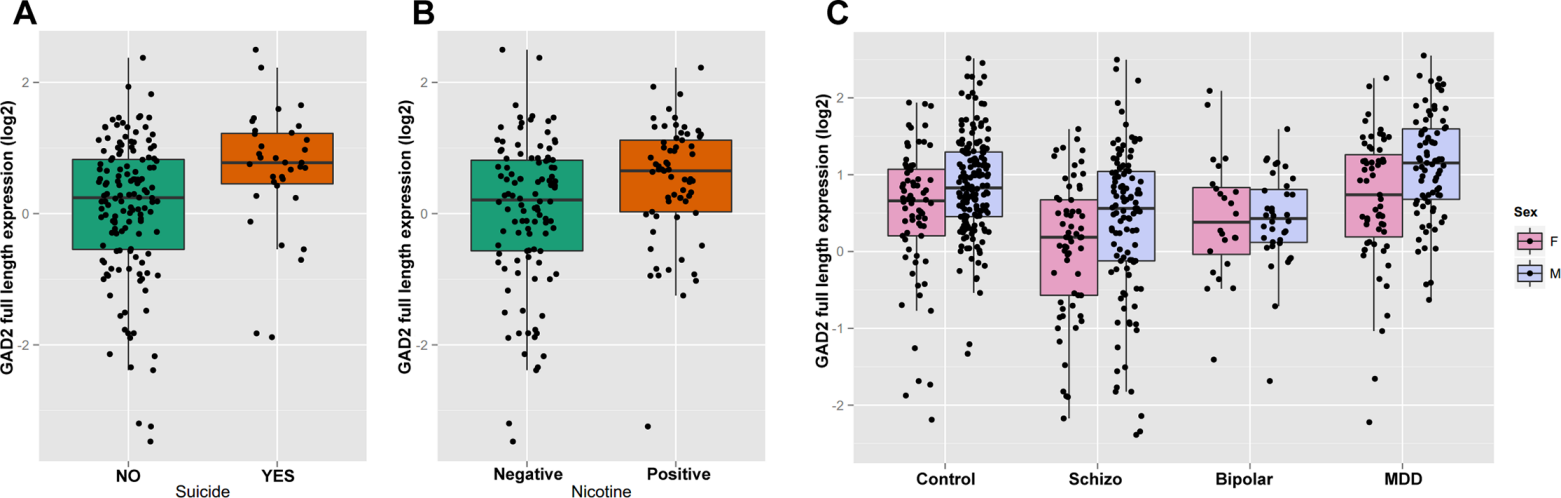
Expression of *GAD2* full length transcript correlated with suicide, nicotine or sex. The y-axis represents relative expression in the DLPFC. The y-axis is the least-squares means of expression with log2 normalization, computed for covariates at their means. Each dot represents an individual subject. (A) *GAD2* full length expression in DLPFC between completed suicide and death by natural causes in schizophrenia patients. The x-axis represents the schizophrenia patients with or without completed suicide. (B) *GAD2* full length expression in DLPFC between nicotine positive and nicotine free schizophrenia patients on toxicology testing. The x-axis represents the schizophrenia patients with or without nicotine exposure based on toxicology testing. (C) *GAD2* full length expression in DLPFC between female and male subjects. The x-axis shows the different diagnostic groups: Control, nonpsychiatric control group; Schizo, patients with schizophrenia; MDD, patients with major depressive disorder. M represent male, F represent female.

The toxicology screen provided the positive or negative results for a large number of substances including nicotine, ethanol, opiates, anticonvulsants, antidepressants, and antipsychotics. The clinical and medical examiner records provided basic demographic information plus lifetime neuroleptic exposure, average daily dose, final neuroleptic dose, and suicide. The multiple regression analyses revealed that the expression of *GAD2* full length transcript in DLPFC was significantly correlated with completed suicide (p = 9.61E-09) and nicotine exposure (p = 2.59E-02) in the psychiatric patients taken as a whole across diagnoses. The subjects with schizophrenia and completed suicide (p = 2.73E-03, Fig 5A) or positive nicotine exposure (p = 1.87E-03, Fig 5B) showed significantly higher expression of *GAD2* full length transcript compared with natural deaths or nicotine free patients with schizophrenia. As there was a significant correlation between sex and the expression of *GAD2* full length transcript, we ran further analyses to determine if sex might affect the expression of *GAD2* full length transcript in the DLPFC within each diagnostic group. The female control subjects had significantly lower expression of *GAD2* full length transcript compared to male controls (p = 1.73E-03). This was also seen independently in schizophrenia (P = 1.19E-02) and MDD (1.20E-03) patients (Fig 5C). There was no significant effect of psychotropic medications on the expression of *GAD2* transcripts in the DLPFC.

## Discussion

Our results confirmed that *GAD2* produces a truncated transcript in the human brain, in addition to the well-known full length transcript that encodes the 65kDa GAD enzyme. Characterization of the expression of these transcripts across the lifespan in the human DLPFC suggests that they are developmentally regulated. Finally, the expression of both *GAD2* transcripts differed significantly between patients with psychiatric disorders compared to non-psychiatric controls.

### Alternative splicing of *GAD2*

This study is the first to characterize alternative splicing of *GAD2* in the human brain. We confirmed the presence of the full length transcript responsible of *GAD2*, and identified its developmental expression pattern in the prefrontal cortex. The truncated *GAD2* transcript consisting of the first four exons of *GAD2*, ENST00000428517 lacks an in-frame stop codon. While the sequence for ENST00000428517 previously has been deposited into the UCSC Genome Browser, nothing has been published regarding its expression in human brain. Lacking of an in-frame stop codon, the truncated transcript is prone to nonstop decay, a cellular mRNA surveillance mechanism to prevent mRNA without a stop codon from translation [23,24]. As alternative splicing can lead to increased functional diversity in mRNA transcripts [25,26], this truncated *GAD2* splice variant probably works as on-off regulation to adjust the level of the enzymatic protein produced by full length *GAD2* transcript, which has a well established role in GABA synthesis. This truncated transcript might be critical to maintain a low level of full length *GAD2* expression during fetal brain development since its lifespan expression pattern is the inverse of the full length *GAD2* transcript. Additional experiments will be needed in order to define the function of this truncated transcript

### *GAD2* splice variants expression is developmentally regulated

Studies have shown that multiple transcripts derived from genes have expression patterns that differ across the lifespan [27,28]. Chan and colleagues detected the full length *GAD2* mRNA in the human fetal frontal pole at 12 gestational weeks, with expression being highest at the beginning of the second trimester and decreasing rapidly and becoming undetectable by gestational week 19 [29]. However, in this study we show that the expression of the full length *GAD2* mRNA increased with age, and had a particularly rapid rise during the first two decades of life, which mirrors (albeit on a different time scale) with expression studies in the rodent [30,31]. The lifetime expression trajectory of the ENST00000428517 is highest during fetal periods and gradually decreases after birth. Since the truncated transcript will be degraded quickly as it is an mRNA lacking a stop codon, its expression more likely acts as a component of the regulation of full length *GAD2* expression levels during fetal brain development. During early CNS development, GABA levels are tightly controlled, as this neurotransmitter plays a major role in the regulation of cell proliferation, differentiation, and migration [32–34]. The role of GABA in early brain development may be mediated in part by the ENST00000428517 transcript since it is highly expressed during in the second trimester fetus, at a time before the rise in levels of synaptosomal full length *GAD2*.

### Schizophrenia and *GAD2*

Recent studies indicate that genes involved in the etiology of schizophrenia may also be involved in the pathology of bipolar disorder and major depression [28,35–37]. Accordingly we examined the expression of *GAD2* splice variants across these disorders. The full length *GAD2* mRNA was significantly decreased in expression in the DLPFC of patients with schizophrenia and bipolar disorder compared with controls. While a decrease in full length *GAD2* mRNA expression in bipolar disorder has been previously reported in the hippocampus, this same group did not see an effect in schizophrenia [38]. Another group has reported that GAD2 full length protein expression was decreased in the primary auditory cortex and cerebellum of schizophrenia patients [37,39] and cerebellum of bipolar disorder patients [37]. Our finding of decreased full length *GAD2* mRNA potentially reflects an immature level of prefrontal development or regression after onset of illness, a supposition based on the developmental trajectory of *GAD2* full length expression across normal development. Schizophrenia and bipolar disorder not only have some common clinical phenotypes, but appear to share some genetic risk factors [40,41]. It is possible that the decrease of full length *GAD2* expression in both diagnostic groups might be involved in the molecular mechanism of the shared phenotypes. The truncated *GAD2* transcript showed a significant decrease in expression level in patients with schizophrenia and an increase in patients with bipolar disorder, but no difference was detected in MDD patients. The expression level of ENST00000428517 suggests that this transcript behaves differently in the pathology associated with schizophrenia and bipolar disorder. The role of the truncated transcript ENST00000428517 is undefined, but it may be a component of the molecular dysfunction associated with these two psychiatric disorders.

It must be highlighted that any differences in mRNA expression between diagnostic groups are difficult to interpret. It is very hard to disambiguate changes related to the pathophysiology of illness from those secondary to the epiphenomena of illness, such as medications, cigarette smoking, environmental deprivation, and/or the stress of a lifetime combating chronic disability. Regardless, there appears to be diagnostic variation for alternative *GAD2* transcripts in schizophrenia and affective disorders.

Our toxicology screen results suggest, unsurprisingly, that the psychiatric patients in this study have been exposed to multiple substances since positive toxicology was not an exclusion criterion for the patients with a psychiatric disorder. We studied the possibility that exposure to either prescription medications, nicotine, or illicit substances may contribute to expression changes seen in patients. Chronic administration of psychotropic medications, including clozapine, fluoxetine, haloperidol, lithium, olanzapine and valproic acid reportedly alters the level of full length GAD2 and full length GAD1 in rat, measured by both real-time PCR and western blotting [42]. In particular, the mood stabilizer valproic acid stimulated the proliferation of the GABAergic neurons with an associated dramatic increase in levels of GABA and full length GAD1/2 [43]. In contrast to animal studies, our analyses didn’t reveal positive correlations between the expression of *GAD2* transcripts in the DLPFC and psychotropic medications in psychiatric patient cohorts.

The most common psychoactive substance on toxicological screening was nicotine, and this was not an exclusion criterion in any diagnostic group. The schizophrenia patients with nicotine exposure had increased expression of *GAD2* full length transcript in the DLPFC comparing to nicotine-free patients with schizophrenia. There have been no previous studies on the impact of nicotine exposure on the expression of *GAD2* in brain. Further animal studies are needed to confirm this finding and delineate the molecular mechanism. The correlation between *GAD2* full length and nicotine is intriguing and suggests a previously unrecognized role for nicotine in cortical function in schizophrenia. This is especially important given the prevalence of cigarette smoking among schizophrenia patients [44].

43.6% psychiatric patients involved in this study were completed suicide. More than half of patients with bipolar disorder or MDD were suicides. We observed the up-regulated expression of *GAD2* full length transcript in patients with schizophrenia who are suicides. Previously, suicidal patients with mood disorders had an increase in the relative density of GAD-immunoreactive neuropil [45] and abnormalities in the glutamate-glutamine and GABA-glutamine cycles had significant impact on suicidal behavior [46]. Our finding suggests that up-regulated 65kDa GAD, the translated protein produced by the *GAD2* full length transcript, might in part be responsible for the increased level of GAD in suicidal patients with mood disorders, and provides additional and potentially important information regarding the role of GABA on suicidal behavior. If confirmed, this offers a new insight into the neurobiology of suicide.

Full length *GAD2* expression is present in most classes of GABA neurons and is particularly prominent in axon terminals [47]. The data in this study is derived from homogenized tissue, and contains *GAD2* transcripts from a variety of cell types and axon terminals. It is possible that the expression changes of *GAD2* gene are restricted to a particular class of neurons or projections, which requires another level of investigation, which is necessary to understand the expression changes of *GAD2* gene at cellular level in brain.

In general, the findings presented here confirm that splice variants of *GAD2* are expressed differently in the human DLPFC across normal brain development and that these two transcripts may be involved in the pathophysiology of schizophrenia and affective disorders. While our results did show that ENST00000428517 is a fetal predominant transcript, its role in development is unknown. In order to address this issue, it would be insightful to conduct behavioral tests on transgenic ENST00000428517 knockout, knockdown and knock-up mice, followed by molecular biological experiments to observe if there are any changes in brain morphology and neurochemistry. It is known that GAD2 knockout mice are susceptible to seizures, have impairments in plasticity-related tasks, such as ocular dominance plasticity during the critical period and induction of early LTD in the visual cortex [14,16,17,48]. The identification of a fetal predominant novel truncated transcript produced by *GAD2* suggests that this gene may play a more complex role in brain development and function than is widely appreciated.

## References

[1] Luján R, Shigemoto R, López-Bendito G. Glutamate and GABA receptor signalling in the developing brain. Neuroscience. 2005;130: 567–580. 10.1016/j.neuroscience.2004.09.042 15590141

[2] Wassef A, Baker J, Kochan LD. GABA and schizophrenia: a review of basic science and clinical studies. J Clin Psychopharmacol. 2003;23: 601–640. 10.1097/01.jcp.0000095349.32154.a5 14624191

[3] Taylor SF, Tso IF. GABA abnormalities in schizophrenia: a methodological review of in vivo studies. Schizophr Res. 2015;167: 84–90. 10.1016/j.schres.2014.10.011 25458856 PMC4409914

[4] Walls AB, Nilsen LH, Eyjolfsson EM, Vestergaard HT, Hansen SL, Schousboe A, et al GAD65 is essential for synthesis of GABA destined for tonic inhibition regulating epileptiform activity. J Neurochem. 2010;115: 1398–1408. 10.1111/j.1471-4159.2010.07043.x 21039523

[5] Akbarian S, Huang H-S. Molecular and cellular mechanisms of altered GAD1/GAD67 expression in schizophrenia and related disorders. Brain Res Rev. 2006;52: 293–304. 10.1016/j.brainresrev.2006.04.001 16759710

[6] Bu DF, Erlander MG, Hitz BC, Tillakaratne NJ, Kaufman DL, Wagner-McPherson CB, et al Two human glutamate decarboxylases, 65-kDa GAD and 67-kDa GAD, are each encoded by a single gene. Proc Natl Acad Sci U S A. 1992;89: 2115–2119. 1549570 10.1073/pnas.89.6.2115 PMC48607

[7] Battaglioli G, Liu H, Martin DL. Kinetic differences between the isoforms of glutamate decarboxylase: implications for the regulation of GABA synthesis. J Neurochem. 2003;86: 879–887. 12887686 10.1046/j.1471-4159.2003.01910.x

[8] Hashimoto T, Bazmi HH, Mirnics K, Wu Q, Sampson AR, Lewis DA. Conserved regional patterns of GABA-related transcript expression in the neocortex of subjects with schizophrenia. Am J Psychiatry. 2008;165: 479–489. 10.1176/appi.ajp.2007.07081223 18281411 PMC2894608

[9] Dracheva S, Elhakem SL, McGurk SR, Davis KL, Haroutunian V. GAD67 and GAD65 mRNA and protein expression in cerebrocortical regions of elderly patients with schizophrenia. J Neurosci Res. 2004;76: 581–592. 10.1002/jnr.20122 15114630

[10] Guidotti A, Auta J, Davis JM, Di-Giorgi-Gerevini V, Dwivedi Y, Grayson DR, et al Decrease in reelin and glutamic acid decarboxylase67 (GAD67) expression in schizophrenia and bipolar disorder: a postmortem brain study. Arch Gen Psychiatry. 2000;57: 1061–1069. 11074872 10.1001/archpsyc.57.11.1061

[11] Glausier JR, Kimoto S, Fish KN, Lewis DA. Lower Glutamic Acid Decarboxylase 65-kDa Isoform Messenger RNA and Protein Levels in the Prefrontal Cortex in Schizoaffective Disorder but Not Schizophrenia. Biol Psychiatry. 2014; 10.1016/j.biopsych.2014.05.010 PMC4247819 24993056

[12] Patel AB, de Graaf RA, Martin DL, Battaglioli G, Behar KL. Evidence that GAD65 mediates increased GABA synthesis during intense neuronal activity in vivo. J Neurochem. 2006;97: 385–396. 10.1111/j.1471-4159.2006.03741.x 16539672

[13] Kanaani J, Kolibachuk J, Martinez H, Baekkeskov S. Two distinct mechanisms target GAD67 to vesicular pathways and presynaptic clusters. J Cell Biol. 2010;190: 911–925. 10.1083/jcb.200912101 20805323 PMC2935578

[14] Asada H, Kawamura Y, Maruyama K, Kume H, Ding R, Ji FY, et al Mice lacking the 65 kDa isoform of glutamic acid decarboxylase (GAD65) maintain normal levels of GAD67 and GABA in their brains but are susceptible to seizures. Biochem Biophys Res Commun. 1996;229: 891–895. 8954991 10.1006/bbrc.1996.1898

[15] Asada H, Kawamura Y, Maruyama K, Kume H, Ding RG, Kanbara N, et al Cleft palate and decreased brain gamma-aminobutyric acid in mice lacking the 67-kDa isoform of glutamic acid decarboxylase. Proc Natl Acad Sci U S A. 1997;94: 6496–6499. 9177246 10.1073/pnas.94.12.6496 PMC21078

[16] Hensch TK, Fagiolini M, Mataga N, Stryker MP, Baekkeskov S, Kash SF. Local GABA circuit control of experience-dependent plasticity in developing visual cortex. Science. 1998;282: 1504–1508. 9822384 10.1126/science.282.5393.1504 PMC2851625

[17] Tian N, Petersen C, Kash S, Baekkeskov S, Copenhagen D, Nicoll R. The role of the synthetic enzyme GAD65 in the control of neuronal gamma-aminobutyric acid release. Proc Natl Acad Sci U S A. 1999;96: 12911–12916. 10536022 10.1073/pnas.96.22.12911 PMC23160

[18] Heldt SA, Green A, Ressler KJ. Prepulse inhibition deficits in GAD65 knockout mice and the effect of antipsychotic treatment. Neuropsychopharmacol Off Publ Am Coll Neuropsychopharmacol. 2004;29: 1610–1619. 10.1038/sj.npp.1300468 15114343

[19] Hyde TM, Lipska BK, Ali T, Mathew SV, Law AJ, Metitiri OE, et al Expression of GABA signaling molecules KCC2, NKCC1, and GAD1 in cortical development and schizophrenia. J Neurosci Off J Soc Neurosci. 2011;31: 11088–11095. 10.1523/JNEUROSCI.1234-11.2011 PMC3758549 21795557

[20] Lipska BK, Deep-Soboslay A, Weickert CS, Hyde TM, Martin CE, Herman MM, et al Critical factors in gene expression in postmortem human brain: Focus on studies in schizophrenia. Biol Psychiatry. 2006;60: 650–658. 10.1016/j.biopsych.2006.06.019 16997002

[21] Straub RE, Lipska BK, Egan MF, Goldberg TE, Callicott JH, Mayhew MB, et al Allelic variation in GAD1 (GAD67) is associated with schizophrenia and influences cortical function and gene expression. Mol Psychiatry. 2007;12: 854–869. 10.1038/sj.mp.4001988 17767149

[22] Jaffe AE, Shin J, Collado-Torres L, Leek JT, Tao R, Li C, et al Developmental regulation of human cortex transcription and its clinical relevance at single base resolution. Nat Neurosci. 2015;18: 154–161. 10.1038/nn.3898 25501035 PMC4281298

[23] Frischmeyer PA, van Hoof A, O’Donnell K, Guerrerio AL, Parker R, Dietz HC. An mRNA surveillance mechanism that eliminates transcripts lacking termination codons. Science. 2002;295: 2258–2261. 10.1126/science.1067338 11910109

[24] van Hoof A, Frischmeyer PA, Dietz HC, Parker R. Exosome-mediated recognition and degradation of mRNAs lacking a termination codon. Science. 2002;295: 2262–2264. 10.1126/science.1067272 11910110

[25] Graveley BR. Alternative splicing: increasing diversity in the proteomic world. Trends Genet TIG. 2001;17: 100–107. 11173120 10.1016/s0168-9525(00)02176-4

[26] Kelemen O, Convertini P, Zhang Z, Wen Y, Shen M, Falaleeva M, et al Function of alternative splicing. Gene. 2013;514: 1–30. 10.1016/j.gene.2012.07.083 22909801 PMC5632952

[27] Colantuoni C, Lipska BK, Ye T, Hyde TM, Tao R, Leek JT, et al Temporal dynamics and genetic control of transcription in the human prefrontal cortex. Nature. 2011;478: 519–523. 10.1038/nature10524 22031444 PMC3510670

[28] Tao R, Li C, Newburn EN, Ye T, Lipska BK, Herman MM, et al Transcript-specific associations of SLC12A5 (KCC2) in human prefrontal cortex with development, schizophrenia, and affective disorders. J Neurosci. 2012;32: 5216–5222. 10.1523/JNEUROSCI.4626-11.2012 22496567 PMC3752043

[29] Chan SO, Lyman WD, Chiu FC. Temporal and spatial expression of glutamic acid decarboxylases in human fetal brain. Brain Res Mol Brain Res. 1997;46: 318–320. 9191108 10.1016/s0169-328x(97)00031-4

[30] Greif KF, Erlander MG, Tillakaratne NJ, Tobin AJ. Postnatal expression of glutamate decarboxylases in developing rat cerebellum. Neurochem Res. 1991;16: 235–242. 1780026 10.1007/BF00966086

[31] Popp A, Urbach A, Witte OW, Frahm C. Adult and embryonic GAD transcripts are spatiotemporally regulated during postnatal development in the rat brain. PloS One. 2009;4: e4371 10.1371/journal.pone.0004371 19190758 PMC2629816

[32] LoTurco JJ, Owens DF, Heath MJ, Davis MB, Kriegstein AR. GABA and glutamate depolarize cortical progenitor cells and inhibit DNA synthesis. Neuron. 1995;15: 1287–1298. 8845153 10.1016/0896-6273(95)90008-x

[33] Marty S, Berninger B, Carroll P, Thoenen H. GABAergic stimulation regulates the phenotype of hippocampal interneurons through the regulation of brain-derived neurotrophic factor. Neuron. 1996;16: 565–570. 8785053 10.1016/s0896-6273(00)80075-6

[34] Manent J-B, Represa A. Neurotransmitters and brain maturation: early paracrine actions of GABA and glutamate modulate neuronal migration. Neurosci Rev J Bringing Neurobiol Neurol Psychiatry. 2007;13: 268–279. 10.1177/1073858406298918 17519369

[35] Fatemi SH, Earle JA, McMenomy T. Reduction in Reelin immunoreactivity in hippocampus of subjects with schizophrenia, bipolar disorder and major depression. Mol Psychiatry. 2000;5: 654–663, 571. 11126396 10.1038/sj.mp.4000783

[36] Costa E, Davis JM, Dong E, Grayson DR, Guidotti A, Tremolizzo L, et al A GABAergic cortical deficit dominates schizophrenia pathophysiology. Crit Rev Neurobiol. 2004;16: 1–23. 15581395 10.1615/critrevneurobiol.v16.i12.10

[37] Fatemi SH, Hossein Fatemi S, Stary JM, Earle JA, Araghi-Niknam M, Eagan E. GABAergic dysfunction in schizophrenia and mood disorders as reflected by decreased levels of glutamic acid decarboxylase 65 and 67 kDa and Reelin proteins in cerebellum. Schizophr Res. 2005;72: 109–122. 10.1016/j.schres.2004.02.017 15560956

[38] Heckers S, Stone D, Walsh J, Shick J, Koul P, Benes FM. Differential hippocampal expression of glutamic acid decarboxylase 65 and 67 messenger RNA in bipolar disorder and schizophrenia. Arch Gen Psychiatry. 2002;59: 521–529. 12044194 10.1001/archpsyc.59.6.521

[39] Moyer CE, Delevich KM, Fish KN, Asafu-Adjei JK, Sampson AR, Dorph-Petersen K-A, et al Reduced glutamate decarboxylase 65 protein within primary auditory cortex inhibitory boutons in schizophrenia. Biol Psychiatry. 2012;72: 734–743. 10.1016/j.biopsych.2012.04.010 22624794 PMC3465514

[40] Cross-Disorder Group of the Psychiatric Genomics Consortium. Identification of risk loci with shared effects on five major psychiatric disorders: a genome-wide analysis. Lancet Lond Engl. 2013;381: 1371–1379. 10.1016/S0140-6736(12)62129-1 PMC3714010 23453885

[41] Cross-Disorder Group of the Psychiatric Genomics Consortium, Lee SH, Ripke S, Neale BM, Faraone SV, Purcell SM, et al Genetic relationship between five psychiatric disorders estimated from genome-wide SNPs. Nat Genet. 2013;45: 984–994. 10.1038/ng.2711 23933821 PMC3800159

[42] Fatemi SH, Reutiman TJ, Folsom TD. Chronic psychotropic drug treatment causes differential expression of Reelin signaling system in frontal cortex of rats. Schizophr Res. 2009;111: 138–152. 10.1016/j.schres.2009.03.002 19359144

[43] Laeng P, Pitts RL, Lemire AL, Drabik CE, Weiner A, Tang H, et al The mood stabilizer valproic acid stimulates GABA neurogenesis from rat forebrain stem cells. J Neurochem. 2004;91: 238–251. 10.1111/j.1471-4159.2004.02725.x 15379904

[44] Kelly DL, McMahon RP, Wehring HJ, Liu F, Mackowick KM, Boggs DL, et al Cigarette smoking and mortality risk in people with schizophrenia. Schizophr Bull. 2011;37: 832–838. 10.1093/schbul/sbp152 20019128 PMC3122289

[45] Gos T, Günther K, Bielau H, Dobrowolny H, Mawrin C, Trübner K, et al Suicide and depression in the quantitative analysis of glutamic acid decarboxylase-Immunoreactive neuropil. J Affect Disord. 2009;113: 45–55. 10.1016/j.jad.2008.04.021 18538859

[46] Bernstein H-G, Tausch A, Wagner R, Steiner J, Seeleke P, Walter M, et al Disruption of glutamate-glutamine-GABA cycle significantly impacts on suicidal behaviour: survey of the literature and own findings on glutamine synthetase. CNS Neurol Disord Drug Targets. 2013;12: 900–913. 24040807 10.2174/18715273113129990091

[47] Esclapez M, Tillakaratne NJ, Kaufman DL, Tobin AJ, Houser CR. Comparative localization of two forms of glutamic acid decarboxylase and their mRNAs in rat brain supports the concept of functional differences between the forms. J Neurosci Off J Soc Neurosci. 1994;14: 1834–1855. 10.1523/JNEUROSCI.14-03-01834.1994 PMC6577546 8126575

[48] Choi S-Y, Morales B, Lee H-K, Kirkwood A. Absence of long-term depression in the visual cortex of glutamic Acid decarboxylase-65 knock-out mice. J Neurosci Off J Soc Neurosci. 2002;22: 5271–5276. 20026507 10.1523/JNEUROSCI.22-13-05271.2002 PMC6758224 12097476

